# Leveraging Microbes for Sustainable Development Goal 14 Progress

**DOI:** 10.1111/1758-2229.70399

**Published:** 2026-08-11

**Authors:** Reshum Aurora, John Paul Balmonte

**Affiliations:** ^1^ Ashland High School Ashland Massachusetts USA; ^2^ Department of Earth and Environmental Sciences Lehigh University Bethlehem Pennsylvania USA

**Keywords:** aquaculture, coastal restoration, marine microbiology, pollutant removal, SDG 14

## Abstract

To promote sustainable use of marine resources, the United Nations Sustainable Development Goal 14 (SDG 14): Life Below Water aims to alleviate the threats of marine pollution, habitat degradation, unsustainable fishing and ocean acidification on ocean ecosystems. With the 2030 deadline approaching, much work remains, necessitating innovative solutions for ocean health and sustainability that can be upscaled and implemented globally. The metabolic versatility of microorganisms makes them powerful, yet currently underutilized tools that can be leveraged to advance SDG 14. Here, we synthesize current microbial technologies that align with primary targets of SDG 14: plastic reduction (14.1), nutrient removal (14.1), coastal restoration (14.2) and sustainable aquaculture (14.7). Diverse microbial groups are involved in these functions in natural and human‐perturbed ecosystems, including plastic‐degrading bacteria, denitrifying communities, host‐associated microbes, biofloc and periphyton systems, as well as photosynthetic microorganisms. We emphasize the ways in which wide‐scale implementation requires mechanistic and systems‐level understanding of microbial applications, environmental testing in variable environments and the development of monitoring and deployment infrastructure. We also highlight the importance of protecting microbial biodiversity to preserve the ecosystem services that will maintain the outcomes of SDG 14. Integrating microorganisms into existing ocean management and policy structures represents a compelling pathway to accelerate progress towards SDG 14—a conceptual framework that can be applied to other SDGs.

## Graphical abstract

Microbial systems can contribute to UN Sustainable Development Goal 14: Life Below Water efforts by transforming plastics, removing excess nitrogen, supporting coastal restoration and improving aquaculture sustainability. Advancing these applications requires field‐scale validation, ecological risk assessment and innovative deployment strategies.

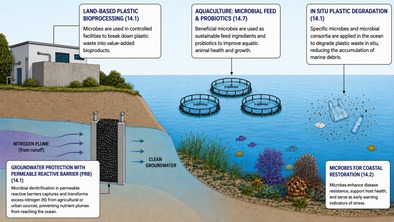

## 1 Introduction

Microorganisms underpin the biogeochemical and ecological processes that sustain our planet. As pressures from industrialization and resource use intensify, ocean ecosystems will continue to face intersecting challenges, including marine pollution, coastal habitat degradation and ocean acidification (Halpern et al. 2008). These stressors affect marine biodiversity, but also human livelihoods, food security and economic stability (Worm et al. 2006).

Recognizing the importance of ocean health, the United Nations adopted Sustainable Development Goal 14 (SDG 14): Life Below Water, as part of the 17 Sustainable Development Goals issued in 2016, with a target year of 2030 for completion. SDG 14 calls for the conservation and sustainable use of oceans and marine resources, comprising multiple targets to achieve marine pollution reduction, coastal restoration and economic resilience for coastal communities, among others. While meaningful progress has been made towards SDG 14, significant gaps remain until the 2030 goal. For example, the number of stations to monitor marine pollution has grown from 2021 to 2025; however, nutrient pollution and ocean acidification continue to intensify (United Nations 2025). In coastal protection and restoration efforts, more countries have been engaged in marine spatial planning. Marine protected areas now cover 8.4% of the ocean but still fall short of the 30% target by 2030 (United Nations 2025). For sustainable fisheries and coastal economies, international cooperation against illegal fishing has improved from a medium to high implementation rating. However, illegal, unreported and unregulated fishing still accounts for up to 15% of global catches, continuing to undermine the food security and livelihoods of coastal communities (United Nations 2025). Collectively, these gaps demonstrate the progress and challenges for meeting SDG 14 targets, with substantial areas of growth required, particularly towards reduction of marine pollution (target 14.1), protection and restoration of ecosystems (target 14.2) and increasing the economic benefits from sustainably using marine resources (target 14.7).

Here, we develop the perspective that microbes are powerful, yet underutilized tools that can be leveraged for pollutant degradation, excess nutrient removal, coastal restoration efforts and enhancement of food webs for sustainable aquaculture to advance efforts towards SDG 14. Owing to their metabolic versatility, microorganisms can serve as direct interventions or support existing management strategies in a wide range of settings and applications (Akinsemolu 2018; Crowther et al. 2024; Fagunwa and Olanbiwoninu 2020). Microbial genomic potentials are well characterized in a range of marine environments, but a critical gap remains in mechanistic and systems‐level understanding needed to define microbial limits and predict behaviour in complex environments. To effectively adopt microbes as SDG 14 tools, verification of microbial metabolic pathways and interactions is critical, especially in real‐world conditions and adjacent management infrastructure needs to be designed. We synthesize current knowledge on microbial applications and outline key research pathways and implementation strategies towards SDG 14, as summarized in Table 1. Finally, we advocate for microbial conservation and stewardship in the ocean, an emerging idea gaining traction across sectors (Choudoir et al. 2025; Gilbert et al. 2025; Junker and Farwig 2025). We anticipate that framing microbial processes and properties as potential solutions also advances progress towards target 14.8, which is to increase scientific knowledge, research and technology to improve and sustain ocean health.

**TABLE 1:** List of select microbial species and functions that can be leveraged for SDG 14 solutions.

Microbe(s)	Microbial function	References	SDG 14 contribution	SDG 14 target
*Ideonella sakaiensis* 201‐F6	Production of PETase and MHETase to break down PET plastic	Yoshida et al. (2016)	Plastic degradation	14.1
*Cupriavidus necator* H16	Intracellular synthesis and accumulation of PHB through the *phaCAB* pathway	Zhang et al. (2022)	Production of biodegradable plastic alternatives	14.1
Woodchip‐associated denitrifying microbes: *Cellulomonas cellasea* WB94 and *Microvirgula aerodenitrificans* BE2.4	Conversion of nitrate to gaseous nitrogen	Hiller et al. (2015)	Interception of nitrogen pollution before coastal discharge, applied in permeable reactive barriers	14.1
*Chlorella vulgaris*	Assimilation of dissolved inorganic nitrogen and phosphorus during biomass growth	Ruiz‐Marin et al. (2010)	Interception of nitrogen pollution before coastal discharge, applied in wastewater systems	14.1
Coral pBMC consortium: five *Pseudoalteromonas* strains, *Halomonas taeanensis* and a *Cobetia marina* –related strain	Reactive oxygen species detoxification, pathogen inhibition and nutrient cycling	Rosado et al. (2019)	Coastal resilience via reduction of coral bleaching under heat stress	14.2
Mangrove plant actinobacterial consortium: *Streptomyces coelicoflavus* UAE2, *S. polychromogenes* UAE1, *S. bacillaris* UAE1 and *S. ferrugineus* UAE1	Phosphate solubilization and production of growth promoting compounds	El‐Tarabily et al. (2021)	Promotion of *Avicennia marina* seedling establishment and growth to support mangrove restoration	14.2
*Lactiplantibacillus plantarum* AH 78	Colonization of the fish intestinal lining and stimulation of fish immune defences	Hamdan et al. (2016)	Aquaculture probiotic: improve fish health, disease resistance and production efficiency	14.7
*Limnospira platensis* (formerly *Arthrospira platensis*)	Production of protein, antioxidant and pigment rich biomass	Mahmoud et al. (2018)	Aquaculture feed: improve fish growth, feed efficiency and reduce fishmeal reliance	14.7

## 2 SDG 14.1 – Reduction of Marine Debris

SDG 14.1 calls for a reduction in marine debris, in part amid an estimated 9–14 million metric tons of plastic that enter the ocean annually (Reddy and Lau 2020). Dominant polymers such as polyethylene terephthalate (PET), polyethylene (PE), polypropylene (PP), polyvinyl chloride (PVC), polystyrene (PS) and polyamide (PA) persist in global oceans due to their structural recalcitrance, leading to long‐term accumulation and ecological harm (Guo and Wang 2019; Thompson et al. 2004; Ahmad et al. 2025; Taylor et al. 2016).

Microbial interventions represent compelling strategies for marine plastic reduction, as microbial enzymes can cleave polymer backbones and metabolize breakdown products (Mohanan et al. 2020; Sharma et al. 2022; Choi et al. 2023). This process can occur naturally in the oceans or be applied in land‐based facilities; however, such scalability first requires addressing incomplete degradation of polymers. For example, some PET‐targeting cutinases achieve only partial degradation, while other studies show that observed reductions in plastic molecular weight may result from weight loss of chemical additives rather than true polymer backbone breakdown (Carniel et al. 2017; Cacciari et al. 1993). To address this limitation, engineered microbial consortia capable of degrading complex polymers via multiple metabolic pathways could be designed (Danso et al. 2019; Choi et al. 2023). A necessary step for implementation is testing microbial plastic degradation under varying in situ conditions, where nutrient availability, temperature and ecological interactions differ from laboratory controls (Sauret et al. 2016). Evaluating limitations under these conditions is critical to ensure reliable degradation outcomes and especially relevant considering natural microbial reactions typically occur at temperatures well below those implemented in biodegradation testing standards.

Once microbial mechanisms are optimized, deployment strategies could be differentiated by plastic size and environmental context. In situ applications could target dispersed microplastics that cannot be removed through physical clean‐up, although delivery methods and ecosystem impacts require further investigation (Scott et al. 2025). Meanwhile, larger plastic debris could be directed towards land‐based bioprocessing systems (Tournier et al. 2020), where controlled conditions could enable efficient degradation. This hybrid model provides a potential roadmap for reducing plastic debris.

Beyond plastic degradation, microbes may also enable valorization pathways, in which plastic‐derived substrates are converted into higher‐value chemical products, such as paracetamol through chemo‐biological processes (Johnson et al. 2025), creating potential economic incentives for plastic removal. Such upstream waste interception has already been demonstrated. For example, a biotechnology company (Carbios) uses engineered microbe‐derived enzymes to depolymerize PET into its monomers under controlled conditions, illustrating the industrialization of microbial applications.

Finally, microbial capabilities can also be leveraged to reduce plastic inputs through the production of biodegradable alternatives. Startup companies such as Mango Materials have utilized microbial processes to convert methane into polyhydroxyalkanoates (Behera and Das 2023). Another approach applies photosynthetic microalgae as renewable feedstocks for bioplastic production. Unlike conventional feedstocks that rely on arable land, microalgae can be cultivated using wastewater and other waste‐derived nutrients, thereby reducing competition with food crops and making them a more resource‐efficient option for bioplastic production (Chia et al. 2020). Scaling these strategies will depend on advances in (1) microbial degradation ability through consortia or genetic engineering, (2) testing in ocean environments and (3) development of in situ deployment methods alongside expansion of land‐based systems.

## 3 SDG 14.1—Reduction of Nutrient Pollution

SDG 14.1 also aims to reduce nutrient pollution from land‐based activities which can be addressed by harnessing microbial capabilities to perform denitrification. In groundwater‐dominated coastal systems, which account for a substantial proportion of coastal zones globally, nitrogen loading can reach 71%–97% of total coastal nitrogen inputs (Valiela et al. 1997; Bowen et al. 2007), making nitrate interception prior to coastal discharge a viable SDG 14.1 strategy. Permeable reactive barriers (PRBs) amended with solid phase carbon, such as woodchips or straw, can be installed in groundwater flow paths or subsurface locations to alleviate electron donor limitation and maintain low‐oxygen conditions, allowing increased denitrification by microbes before nitrogen plumes reach oceans (Hiller et al. 2015). With over 200 global installations since the first full‐scale deployment in 1994 (ITRC 2011) and design lifespans estimated at 10–30 years (Singh et al. 2023), PRBs represent field‐validated groundwater remediation technologies that ensure low nitrate inputs into coastal systems.

A key barrier to the widespread deployment of PRBs is reliably characterizing subsurface flow, as uncertainties remain with regards to nutrient plume geometry and plume residence time variability (Gibert et al. 2019; ITRC 2011). Since incomplete nutrient plume capture is a documented limitation, effectiveness of this strategy will depend on the ability to track underground water movement to improve nutrient interception. Increased monitoring and parallel design changes may help ensure more effective installations. These advances could enable more predictable hydraulic performance to unlock broader application of PRBs as microbial metabolism‐based strategies for intercepting and removing land‐derived nitrogen, making them a viable strategy for reducing coastal eutrophication under SDG 14.1.

Alongside denitrifying PRBs, microalgae and cyanobacteria can be leveraged in wastewater treatment as another route for preventing excess nutrient inputs in coastal waters. These organisms can assimilate inorganic carbon, nitrogen, phosphorus and micronutrients into biomass in wastewater (El‐Sheekh et al. 2022). Harvested microalgae may subsequently be converted into high‐value compounds such as biohydrogen and bioalcohols (Hashemian et al. 2019). Broader implementation of this strategy depends on developing cultivation systems that maintain consistent nutrient‐removal performance despite variations in wastewater composition and light availability (Abdelfattah et al. 2023). Once these limitations are addressed, photosynthetic microorganisms could provide more energy‐efficient nutrient removal in wastewater treatment systems, potentially lowering operating costs compared to conventional biological treatment methods (Abdelfattah et al. 2023; El‐Sheekh et al. 2022).

## 4 SDG 14.2—Protection and Restoration of Ecosystems

Coastal ecosystems, including coral reefs, seagrass meadows, mangroves, salt marshes, dunes and macroalgal forests, are foundational to numerous endemic species, global biogeochemical cycles and human livelihoods. Approximately 38% of the global population lives within 100 km of coastlines (Kummu et al. 2016) and depend on these systems for food security, storm protection, carbon sequestration and pollutant filtration (Marine and Coastal Ecosystems and Human Well‐Being 2006). Climate change, pollution, habitat conversion and sea‐level rise continue to place pressure on these systems. In response, SDG 14.2 calls for protection and restoration to enhance ecosystem resilience. However, transplantation‐based strategies, the dominant current approach to restore these ecosystems, are constrained by high post‐transplant mortality and incomplete recovery at many sites (Duarte et al. 2015).

Applying the holobiont concept to restore foundational coastal species recognizes the need to consider microorganisms as integral components of any macroorganism that are responsible for providing nutrients, disease resistance and stress tolerance (Van Oppen et al. 2009; Tarquinio et al. 2019). Therefore, incorporating microbes into restoration plans may promote more transplantation successes. Probiotic inoculations, for example, have reduced coral disease by suppressing pathogens including *Serratia marcescens* (Ritchie 2006; Alagely et al. 2011) and microbiome manipulation has improved thermal tolerance and reduced bleaching severity in transplanted corals (Epstein et al. 2019; Doering et al. 2021). In seagrasses, sulfide‐oxidizing bacteria can remove toxic hydrogen sulfide from sediments, improving host survival in degraded sites (Ugarelli et al. 2017). Thus, microbial probiotics have substantial potential to enhance transplantation across coastal ecosystems. Additionally, microbes can also function as early warning indicators in coastal restoration. In corals, microbial community shifts towards dysbiosis can precede visible bleaching or disease (Van De Water et al. 2016). Hence, we suggest integrating microbiome diagnostics into restoration practices, allowing targeted action before large‐scale mortality occurs.

Several pathways and targeted efforts could enable field deployment by addressing three key constraints. First, introduced consortia may fail to establish long‐term residence within host‐associated or environmental microbiomes due to competition, dispersal and environmental variability (Peixoto et al. 2017). Establishing controlled delivery systems of microbial consortia will therefore be essential for microbe‐integrated coastal restoration. Initial deployment approaches, such as carrier‐mediated transport (Assis et al. 2020) and repetitive in situ dosing (Delgadillo‐Ordoñez et al. 2024), should continue to be explored and tested. Second, microbial function is highly context‐dependent, with probiotic efficacy varying across environmental gradients, such as temperature, nutrient availability and host condition (Peixoto et al. 2017). Validating probiotic efficacy across transplantation environments and standardizing microbiome monitoring protocols will be important to scale microbial restoration strategies. Third, microbiome interventions may alter existing microbial networks, potentially reshaping nutrient cycling pathways or displacing native taxa. Long‐term ecological consequences remain uncertain and must therefore be investigated (Delgadillo‐Ordoñez et al. 2024; Lensch et al. 2024). The use of native or site‐adapted microbial consortia is suggested as a path forward because they are more likely to persist and naturally integrate into existing microbial communities (Peixoto et al. 2017). Overall, microbe‐assisted restoration can be a powerful approach to bolster coastal restoration and research for ecological validation and targeted applications must continue to address SDG 14.2.

## 5 SDG 14.7—Increase Economic Benefits From Sustainable Use of Marine Resources

SDG 14.7 seeks to strengthen economic returns to Small Island Developing States and least developed countries through the sustainable use and management of marine resources, including fisheries and aquaculture. Aquaculture systems commonly face accumulation of nitrogenous waste, particularly ammonia from feed inputs and organismal excretion, alongside disease outbreaks and low feed conversion efficiency that limit productivity and sustainability (Martínez‐Córdova et al. 2015). In principle, heterotrophic microbes in these waters can assimilate dissolved inorganic nitrogen, including ammonia, into microbial biomass, thereby reducing nitrogen concentrations and sustaining healthy aquaculture systems (Avnimelech 1999; Ebeling et al. 2006). However, these processes may also be operationalized in biofloc and periphyton‐based systems, where microbial consortia simultaneously provide nutrient supplements as feed and water treatment. Periphyton‐based biofilms, in particular, have been shown to reduce formulated feed inputs by up to 60% while maintaining comparable fish growth and biomass production, demonstrating their potential (Milstein et al. 2008). Microalgae are also promising for this purpose, supporting larval and juvenile fish production as live feed, either directly or through the enrichment of zooplankton, and serving as ingredients in formulated feeds that may reduce reliance on fishmeal and fish oil (Thépot et al. 2016; Sarker et al. 2020). When cultivated within rearing water or treatment units, microalgae can assimilate excess nitrogen and phosphorus (Siddik et al. 2024; Raza et al. 2025). Microorganisms therefore function as both nutrient cyclers and food sources that support productive aquaculture systems (Martínez‐Córdova et al. 2015). Similarly, probiotic microorganisms may enhance digestion, nutrient utilization, immune responses and pathogen resistance in fish, leading to improved growth and feed conversion efficiency (EL‐Haroun et al. 2006; Bricknell and Dalmo 2005).

A primary constraint to scaling microbial aquaculture technologies is high environmental context‐dependence of outcomes, as performance depends strongly on factors such as microbial strain, dosing, environmental conditions and system management (Hai 2015). In addition, operational demands, such as reliable aeration, water‐quality monitoring and technical expertise, limit adoption of these approaches, particularly in low‐resource and small‐scale systems (Emerenciano et al. 2021). To overcome these barriers, future efforts should prioritize standardized deployment protocols, improved monitoring systems and environment‐specific validation of microbial solutions (Hai 2015; Fagunwa and Olanbiwoninu 2020). If these barriers are surpassed, microbial applications could increase aquaculture productivity sustainably to support fisheries in underdeveloped countries for SDG 14.7.

## 6 Sustaining Ocean Health Through Microbial Conservation

Microbes offer broad and robust solutions for SDG 14 due to their diversity, metabolic versatility and ecosystem services in the oceans. Following continued research in the field or under environmentally relevant conditions, as well as improvements in scalability, deployment strategies and management, microbial technologies can complement pre‐existing interventions towards achieving ocean sustainability goals. Progress in these research areas ensures the reliability of microbial applications across marine environments.

Advancing these innovations also requires that natural microbial communities remain diverse and healthy in marine systems. A recent push towards microbial conservation will thus be integral to all the environmental applications discussed (Gilbert et al. 2025; Junker and Farwig 2025), as the effectiveness of plastic degradation, nutrient removal, coastal restoration and sustainable aquaculture interventions relies on maintaining the diverse functions of microbial communities. Under healthy and diverse states, marine microbial communities naturally carry out biogeochemical processes and ecosystem services that will sustain the outcomes of SDG 14. Therefore, protecting microbial biodiversity is essential for natural ecosystem resilience and to continue research and discovery of microbial attributes that can be leveraged to maintain ocean health. Incorporating microbes into environmental policy frameworks can enhance research and conservation priorities that advance SDG 14 and the collective sustainable development goals.

## Author Contributions

**Reshum Aurora:** conceptualization, writing – original draft, visualization, writing – review and editing. **John Paul Balmonte:** writing – review and editing, conceptualization.

## Funding

This work was supported by National Science Foundation (OCE‐2241721) and Lehigh Oceans Research Center.

## Conflicts of Interest

The authors declare no conflicts of interest.

## Data Availability

Data sharing not applicable to this article as no datasets were generated or analysed during the current study.
